# Pernicious Anemia Unveiled: Unusual Hemolytic Complications and Clinical Implications

**DOI:** 10.7759/cureus.57901

**Published:** 2024-04-09

**Authors:** Meesha Trivedi, Aruni Areti, Nikit Venishetty, Amish Parikh, Claudia Didia

**Affiliations:** 1 Internal Medicine, Paul L. Foster School of Medicine, Texas Tech University Health Sciences Center El Paso, El Paso, USA; 2 Orthopedic Surgery, Paul L. Foster School of Medicine, Texas Tech University Health Sciences Center El Paso, El Paso, USA; 3 Internal Medicine, Huntington Hospital, Pasadena, USA

**Keywords:** autoimmune gastritis, pseudo-thrombotic microangiopathy, rare cause of vitamin b12 deficiency, hemolytic anemia, pernicious-anemia

## Abstract

Pernicious anemia (PA) is an autoimmune condition resulting in impaired vitamin B12 absorption that commonly presents with gastritis and neurological symptoms. In rare cases, associated vitamin B12 deficiency can contribute to significant red blood cell lysis, and patients can present with PA-induced pseudo-thrombotic microangiopathy (TMA) hemolytic anemia. This case describes a 59-year-old male presenting with a two-week history of gastrointestinal pain with bleeding who had anemia and hemodynamic instability on initial evaluation. After the endoscopy/colonoscopy did not reveal any active sources of bleeding and packed red blood cells failed to stabilize the patient, it was found that he had low serum B12 with anti-intrinsic factor and anti-parietal cell antibodies. A coordinated clinical approach, including parenteral cyanocobalamin and daily oral folic acid supplementation, stabilized the patient, highlighting the importance of distinguishing PA-induced pseudo-TMA from true TMA hemolytic anemia.

## Introduction

Pernicious anemia (PA) is an autoimmune condition characterized by impaired absorption of dietary vitamin B12 (cobalamin). Vitamin B12 absorption is normally facilitated by the stomach’s parietal cells, which secrete hydrochloric acid (which helps release vitamin B12 from food) and intrinsic factor (IF) (which is required for the absorption of vitamin B12 at the terminal ileum). PA’s autoimmune processes are characterized by the production of two major types of antibodies: anti-IF antibodies and anti-parietal cell antibodies. The autoimmune-mediated destruction causes atrophic gastritis, gradually contributing to achlorhydria, then vitamin B12 deficiency, and then megaloblastic anemia [1,2]. Globally, PA predominantly affects individuals between 60 and 70 years old, with an incidence rate of approximately 2% [2]. Current therapeutic approaches primarily involve vitamin B12 replacement, either orally or parenterally, to address the deficiency and alleviate symptoms [2,3].

Vitamin B12’s critical roles include acting as a cofactor for enzymes necessary in DNA synthesis, fatty acid metabolism, and the formation of the nerve-protecting myelin sheath [3]. B12 is a coenzyme for methionine synthase, an enzyme vital for DNA synthesis in red blood cell precursors [4]. Lack of B12 leads to defective erythropoiesis, marked by the production of large, dysfunctional megaloblastic red cells [4]. B12’s intricate absorption pathway renders it susceptible to disruptions, and deficiencies can manifest with a spectrum of symptoms, encompassing both neurological and hematological disturbances [3,4].

Our case offers a novel exploration into an infrequent association between PA and hemolytic anemia. Literature on this intersection remains sparse, with only a handful of studies, such as that by Andrès et al., pinpointing a mere 1.5% of vitamin B12-deficient patients manifesting with hemolytic anemia [5]. We present a compelling case of a 59-year-old male diagnosed with atrophic gastritis-induced PA who exhibited unusual symptoms of hemolytic anemia, broadening the existing understanding of potential clinical presentations of PA.

## Case presentation

A 59-year-old male presented with a two-week history of constipation and rectal pain with bleeding. He also reported nausea, non-bloody and non-bilious vomiting, poor appetite, palpitations, shortness of breath, and fatigue. Notably, the patient had a history of chronic alcohol use disorder.

Upon admission, the patient’s diagnostic workup revealed severe anemia, indicated by critically low platelet count (PLT), hematocrit, hemoglobin (Hb), critically high lactate dehydrogenase (LDH) levels, and suspected gastrointestinal bleeding (Table 1). Accordingly, the patient received a transfusion of two units of packed red blood cells, which increased and stabilized Hb at 6.6 g/dL. He was given a continuous intravenous infusion of a proton pump inhibitor and octreotide (somatostatin analog). Prophylactic intravenous ceftriaxone was also started.

**Table 1:** Diagnostic workup AST, aspartate aminotransferase; EO ABS #, eosinophil absolute number; Hb, hemoglobin; HCT, hematocrit; INR, international normalized ratio; LDH, lactate dehydrogenase; LYMPH ABS #, lymphocyte absolute number; MCH, mean corpuscular hemoglobin; MCHC, mean corpuscular hemoglobin concentration; MCV, mean corpuscular volume; MONO ABS #, monocyte absolute number; MPV, mean platelet volume; NEUT ABS #, neutrophil absolute number; PLT, platelet count; RBC, red blood cell; RDW-CV, red cell distribution width - CV; RDW-SD, red cell distribution width - SD; Tbil, total bilirubin; WBC, white blood cell

Lab test	Result	Status	Normal range
WBC	7.41 × 10³/µL	Normal	4.5-11.0 × 10³/µL
RBC	1.1 × 10⁶/µL	Low	4.7-6.1 × 10⁶/µL
Hb pre-first transfusion	4.4 g/dL	Critical	13.8-17.2 g/dL
Hb post-first transfusion	6.6 g/dL	Low	13.8-17.2 g/dL
HCT	13%	Critical	40.7-50.3%
MCV	118.2 fL	High	80-96 fL
MCH	40 pg	High	27-33 pg
MCHC	33.8 g/dL	Normal	32-36 g/dL
PLT	179 × 10³/µL	Normal	150-450 × 10³/µL
MPV	12.8 fL	High	7.4-10.4 fL
RDW-CV	25%	High	11.6-14.6%
RDW-SD	78.4 fL	High	39-46 fL
NEUT ABS #	6.22 × 10³/µL	Normal	1.8-7.8 × 10³/µL
LYMPH ABS #	1.19 × 10³/µL	Normal	1.0-4.8 × 10³/µL
MONO ABS #	0 × 10³/µL	Low	0.2-0.8 × 10³/µL
EO ABS #	0 × 10³/µL	Normal	0.0-0.5 × 10³/µL
Heart rate	Up to 120 bpm	High	60-100 bpm
Tbil	3.1 mg/dL	High	0.1-1.2 mg/dL
INR	1.3	High	0.8-1.1
AST	64 U/L	High	0-40 U/L
LDH	2,551	High	120-460 IU/L

The patient underwent esophagogastroduodenoscopy (EGD) and colonoscopy. The EGD revealed gastritis with an atrophic mucosa in the gastric fundus and body, leading to biopsies for further examination. The colonoscopy identified both external and internal hemorrhoids. Subsequent biopsies showed chronic gastritis with marked atrophy and intestinal metaplasia, but no *Helicobacter pylori*, malignancy, dysplasia, or celiac disease. Moreover, the patient was tested for various autoimmune etiologies and other potential causes of anemia (Table 2).

**Table 2:** GI follow-up labs ANA, antinuclear antibody; AMA, anti-mitochondrial antibody; ASMA, anti-smooth muscle antibody

Lab test	Result	Status
Hepatitis panel	Negative	Normal
ANA	Negative	Normal
ASMA	Negative	Normal
AMA	Negative	Normal
Ceruloplasmin	Normal	Normal
Fasting transferrin saturation	Normal	Normal
Ferritin level	Normal	Normal
HFE gene mutation	Negative	Normal
Alpha-1 antitrypsin	Normal	Normal

Additional laboratory results indicated abnormal iron studies, with a low total iron-binding capacity of 256 µg/dL, low transferrin levels at 182 mg/dL, and elevated serum iron at 196 µg/dL. These findings were complemented by a severely low vitamin B12 level (<159 pg/mL) and a high mean corpuscular volume of 118.2 fL, raising suspicion for PA. A direct Coombs test was positive for both IgG and C3, suggesting the presence of warm autoantibodies (Table 3).

**Table 3:** Hematology/oncology follow-up labs IF, intrinsic factor; MCV, mean corpuscular volume; PMN, polymorphonuclear leukocyte; TIBC, total iron-binding capacity

Lab test	Result	Status	Normal range
Hb pre-second transfusion	5.7 g/dL	Low	13.8-17.2 g/dL
Hb post-second transfusion	8.1 g/dL	Low	13.8-17.2 g/dL
MCV	94.8 fL	Normal	80-96 fL
Reticulocyte count	2%	High	0.5-1.5%
TIBC	256 µg/dL	Low	250-450 µg/dL
Transferrin	182 mg/dL	Low	200-360 mg/dL
Ferritin	194 ng/mL	Normal	30-400 ng/mL
Iron	196 µg/dL	High	60-170 µg/dL
% Saturation	77%	High	15-50%
Vitamin B12	<159 pg/mL	Low	232-1245 pg/mL
Direct Coombs test	Positive (IgG and C3)	Abnormal	N/A
IF antibody	Positive	Abnormal	N/A
Antiparietal cell IgG antibody	Positive	Abnormal	N/A
Peripheral blood smear	Hypersegmented PMNs and megalocytes	Abnormal	N/A

A few hours later, the patient’s Hb level dropped again to 5.7 g/dL. This significant and rapid decrease necessitated another red blood cell transfusion and prompted a consultation with hematology/oncology (Table 3). The hematology/oncology team proceeded with additional testing for IF antibodies and anti-parietal cell IgG antibodies, both positive, confirming a diagnosis of PA (Table 3). Furthermore, a peripheral blood smear displayed hypersegmented polymorphonuclear leukocytes and megalocytes, consistent with this diagnosis.

**Figure 1:**
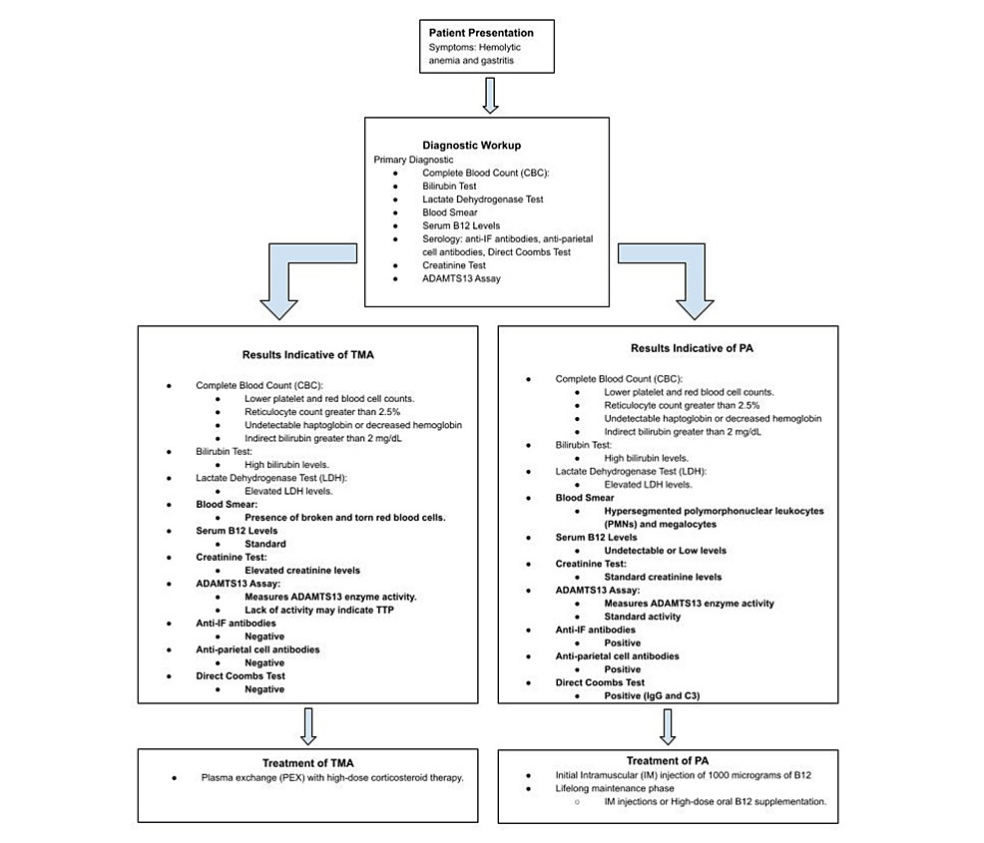
Treatment of the management of PA vs. TMA PA, pernicious anemia; TMA, thrombotic microangiopathy

Due to the patient’s consistent decrease in Hb levels following several transfusions over a few hours, the diagnosis of thrombotic microangiopathy (TMA), a condition unresponsive to plasma exchange that arises due to vitamin B12 deficiency, categorizing it as a secondary manifestation of PA, was made (Figure 1). Furthermore, the patient’s elevated LDH levels suggest hemolytic anemia, potentially stemming from the lysis of red blood cells, a phenomenon that may be attributed to TMA.

Upon establishing the differential diagnosis, the patient was treated with parenteral cyanocobalamin and oral folic acid. This approach successfully managed the vitamin B12 deficiency and anemia, resulting in an increase in the Hb level to 7.0 g/dL.

The patient was discharged with a treatment plan that included parenteral cyanocobalamin and oral folic acid for PA, with scheduled hematology/oncology follow-ups to monitor his Hb and vitamin B12 levels (Figure 1). Coordinated follow-up care for his gastritis and hemorrhoids was established with his primary care provider and gastroenterology.

## Discussion

This case describes a patient with untreated PA who presented with significant gastritis and hemolytic anemia, highlighting pseudo-TMA as a rare but significant hematological complication of PA. Distinguishing TMA secondary to PA from true TMA presents a challenging scenario, particularly given the rarity of pseudo-TMA and the similarities in clinical presentation between these two conditions, like hemolytic anemia, thrombocytopenia, and schistocytosis [6]. In clinical practice, distinguishing between pseudo-TMA due to PA and true TMA is critical because their treatments are different. TMA often requires interventions like plasma exchange and immunosuppression, whereas PA requires vitamin B12 replacement therapy.

Patients with PA-induced vitamin B12 deficiency may suffer from neurological and psychiatric issues, as well as widespread hematologic problems. However, severe hematologic symptoms linked to vitamin B12 deficiency are relatively uncommon [7]. In the study presented by Andrès et al., out of 201 patients with cobalamin deficiency, only 2.5% exhibited pseudo-TMA, and 1.5% presented with hemolytic anemia [5]. When vitamin B12 is deficient, the red blood cell membrane becomes more rigid, leading to increased red blood cell splenic lysis [8]. Furthermore, cobalamin deficiency halts the maturation of all cellular lines within the bone marrow, resulting in hemolytic anemia due to atypical erythropoiesis [9].

In 2022, Abosheaishaa et al. presented a patient exhibiting hemolytic anemia, which led to the suspicion of TMA. However, despite blood transfusions, there was no improvement in Hb levels or PLTs. It was observed that hematological complications showed significant improvement upon correcting low vitamin B12 levels. This underscores the importance of considering PA-associated vitamin B12 deficiency in the differential diagnosis when encountering conditions with similar hematological manifestations. There have been several other cases examining hemolytic anemia in patients with vitamin B12 deficiency [6-13]. In some of these cases, further investigation revealed positive anti-IF antibodies, which confirmed the diagnosis of PA-associated pseudo-TMA [6,8-10].

TMA hemolytic anemia is a serious condition requiring rapid intervention with plasma exchange therapy. Ultimately, correctly identifying PA-associated pseudo-TMA helps avoid unnecessary treatments, reducing the risk of complications from interventions like plasma transfusion, which is necessary for TMA but ineffective for pseudo-TMA. For instance, a study indicated that 34% of patients with anemia were treated with plasma transfusion and/or exchange, but complications secondary to therapy with plasma exchange arose in 14% of these patients [10].

We suggest that in patients with high clinical suspicion for TMA who are presenting with associated gastric or neurological symptomatology, a serum B12 level be added to routine labs to help distinguish TMA from PA-induced pseudo-TMA. Additionally, several studies found that patients with pseudo-TMA have very high LDH levels, a low reticulocyte count, and elevated homocysteine levels, which should prompt screening for PA-associated pseudo-TMA in patients with hemolytic anemia [8,11].

Research has demonstrated that normalization of vitamin B12 levels can be achieved with both oral and intravenous vitamin B12 supplementation. However, due to the severity of anemia, intravenous vitamin B12 administration is often preferred for its rapid efficacy. Considering long-term management and quality of life, oral supplementation may offer a more cost-effective approach following the initial stabilization of the patient’s B12 levels [14].

Given the long biological half-life of body stores of vitamin B12, estimated to be more than 30 months, it is essential to implement a structured follow-up plan for patients. We recommend follow-up appointments at one month, six months, and thereafter annually, to monitor the patient’s blood work and vitamin levels, ensuring sustained improvement and preventing relapses [14].

## Conclusions

Distinguishing between true TMA and PA-induced pseudo-TMA is vital for effective patient management and treatment, particularly in cases presenting with hemolytic anemia. PA often presents with gastritis and vitamin B12 deficiency. Although rare, B12 deficiency can lead to significant red blood cell lysis, resulting in critical hemolytic anemia resembling TMA. Accurate diagnosis is imperative, as it guides the appropriate intervention, including vitamin B12 supplementation, which can reduce or eliminate the need for more invasive treatments like plasmapheresis. However, diagnosing PA is often challenging due to the lack of specific screening tools, standardized testing protocols, and updated guidelines. We suggest that in patients presenting with gastritis and symptoms of hemolytic anemia, healthcare providers retain clinical suspicion for PA-induced TMA. This includes conducting a thorough clinical evaluation and integrating appropriate diagnostic tests to unravel such complex clinical scenarios.
